# 

**Published:** 1887-12

**Authors:** 


CONTENTS.

VOL. 34 NO. 12.

| | PAGE. |
|---|---|
| Last Words | 265 |
| Concerning the Food we Eat | 266 |
| Microbes that Endanger Health | 267 |
| Stand Up for the New and the True | 270 |
| Schools in Medicine | 272 |
| A Case of Trance | 273 |
| What of the Potato | 274 |
| A Three-time Winner | 276 |
| Progress | 277 |
| Book Notices | 278 |
| Household | 280 |
| Miscellaneous | 282 |
| Retrospect | 283 |
| How Ben Butler Got Rich | 284 |

INDEX TO ADVERTISEMENTS OF HALF A PAGE AND OVER.

| | PAGE. |
|---|---|
| Crystal Pepsin Tablets | I |
| Winchester's Hypophosphates | II |
| Zylonite | II |
| Hollow Suppositories | III |
| Celerina | IV |
| Lactopeptine | V |
| Castoria | V |
| Bovinine | VI |
| Crystalline Phosphates | VII |
| Goodyear Rubber Co | VII |
| Telegraph Medicine Co | VIII |
| Dr. Molesworth | VIII |
| J. B. Watkins' Land Mortgage Co | VIII |
| True & Co | X |
| Hallett & Co | X |
| Premiums of Genuine Silver Plate | XI |

